# The antifungal and antibiofilm effects of isobavachalcone on *Candida glabrata* (*Nakaseomyces glabratus*) are associated with intracellular ROS production and cell membrane damages

**DOI:** 10.3389/fcimb.2026.1874660

**Published:** 2026-09-29

**Authors:** Longfei Yang, Yanfeng Zhang, Yu Zhang, Lufei Wang, Xin Liu

**Affiliations:** 1 Jilin Provincial Key Laboratory on Molecular and Chemical Genetics, The Second Hospital of Jilin University, Changchun, China; 2 Department of Blood Transfusion, The Second Hospital of Jilin University, Changchun, China; 3 Department of Clinical Laboratory, The Second Hospital of Jilin University, Changchun, China; 4 Eye Center, The Second Hospital of Jilin University, Changchun, China

**Keywords:** antifungal, biofilm, *Candida glabrata* (*Nakaseomyces glabratus*), cell membrane damage, isobavachalcone (IBC)

## Abstract

Millions of fungal infections are reported every year, while the current antifungal drugs are so few that the clinical choices are limited. The opportunistic fungal pathogen *Candida glabrata* (reclassified as *Nakaseomyces glabratus*) caused more infections than any other *Candida* species except *C. albicans*. This research sought to explore the antifungal effects of isobavachalcone (IBC, a bioactive chalcone from *Psoralea corylifolia*) on *C. glabrata* planktonic growth and biofilms. Our results showed that IBC has potent antifungal activity against *C. glabrata* (MIC: 4-5 μg/mL), including both the standard strain and clinical isolates. This compound could also inhibit *C. glabrata* biofilm formation and development. Its antifungal mechanism involves intracellular ROS overproduction and cell membrane impairments, which were revealed by confocal laser scanning microscope observation after fluorescent staining of ROS and plasma membrane. *N*-acetyl-cysteine rescue tests also confirmed that the ROS-inducing activity of IBC contributed to its antifungal and antibiofilm effects on *C. glabrata*. Micrographs obtained with electron microscope revealed as well the impairments to *C. glabrata* plasma membrane caused by IBC. In conclusion, our results obtained here indicated that IBC is a promising antifungal candidate for treating *C. glabrata* infections.

## 1 Introduction

Among the many fungal pathogens causing severe infections in human, *Candida* species account for a large part. Next to *Candida albicans* (the most prevalent *Candida* pathogen in human), *Candida glabrata* is the second in patients with fungal infections in some countries (Arendrup et al., 2023; Ahamad and Fatma, 2025; Ghosh et al., 2026). This fungus has been reclassified as *Nakaseomyces glabratus* recently, but for some reasons the old name *C. glabrata* was kept in this study. The infections caused by this fungus may occur on surfaces of mucosa and in bloodstreams, the latter of which would endanger life (Ahamad and Fatma, 2025). Biofilm infections associated with medical devices (intrauterine devices (Zahran et al., 2015) and vascular and urinary catheters (d'Enfert and Janbon, 2016)) caused by *C. glabrata* have also been reported. Infections associated with biofilms are notorious for their recalcitrance and the resistance to antifungal drugs. Currently, the antifungal drugs for clinical use are still mainly limited to azoles, polyenes and echinocandins. The fungistatic properties of azoles contribute to the resistance to them, which is a major clinical concern. Although resistance to amphotericin B (AmB, one of polyenes) is rare, its nephrotoxicity makes the clinical use limited. Cell wall-targeting echinocandins are safe in mammals, which makes them recommended as the first-line treatment of *Candida* infections (Dickwella Widanage et al., 2025). Mutation in the hotspot regions of the catalytic subunit of *β* - 1,3-D-glucan synthase (FKS), is the main mechanism of resistance to echinocandins (Liu et al., 2017; Xie et al., 2026). The prevalence of echinocandin resistance in *C. glabrata* is increasing (Jospe‐Kaufman et al., 2024). While the antifungal resistance is increasingly reported and the intrinsically drug-resistant *Candida auris* have already appeared, the current limited armamentarium of antifungal drugs become a great concern (Yang et al., 2024a). In addition, on surfaces of intravascular catheters and dental prosthesis, *C. glabrata* can also form biofilms, which are resistant to azoles and are hard to be eradicated (d'Enfert and Janbon, 2016; Rodrigues et al., 2017). All these call for novel antifungal therapies, especially those effective for *Candida* biofilms.

Natural products from herbal plants attract increasing interest among microbiologists for their diverse pharmacological effects (Liu et al., 2017; Atanasov et al., 2021; Yang et al., 2024a). As one of such small compounds, isobavachalcone (IBC) is one active chalcone firstly isolated from *Psoralea corylifolia* (Bhalla et al., 1968; Wang et al., 2021). It exists also in plants such as *Glycyrrhiza uralensis* (Xiao et al., 2021), *Erythrina burttii* (Yenesew et al., 1998), *Piper longum* (Ohno et al., 2010), *Morus alba* (Yang et al., 2014), and *Kadsura ananosma* (Chen et al., 2006), among others (Xing et al., 2022). IBC has shown antibacterial, antifungal, antiviral, anticancer and anti-inflammatory activities, among others (Wang et al., 2020; He et al., 2021; Zhou et al., 2021; Assis et al., 2022; Yang et al., 2024a). Recent years have seen the increasing reports on the antifungal activities of IBC on fungal pathogens, such as *C. albicans* (Qian et al., 2024b; Wu et al., 2024; Yang et al., 2024a), *Cryptococcus neoformans* (Qian et al., 2024a; Wang et al., 2024), *Magnaporthe oryzae* (Li et al., 2021), *Magnaporthe grisea* (Liu et al., 2018), *Microsporum audorium* (Mbaveng et al., 2008), and *Trichophyton rubrum* (Mbaveng et al., 2008). Although the minimal inhibitory concentration (MIC) of IBC against *C. glabrata* have been reported in one isolate (Mbaveng et al., 2008), the antifungal mechanism of IBC in *C. glabrata* has never been explored. Therefore, through this article, we evaluated the antifungal effects of IBC on *C. glabrata* and explored the antifungal mechanisms.

## 2 Materials and methods

### 2.1 Strains and chemicals

*C. glabrata* ATCC 2001, as well as six *C. glabrata* clinical isolates (the detailed clinical information could be seen in **Supplementary Table 1**), was used in this study. Strains were maintained on yeast extract-peptone-dextrose (YPD) agar in 4 °C fridge. Before each assay, one colony of each strain were transferred into YPD broth for overnight proliferation at 28 °C, with a rotation of 140 rpm. IBC, bought from Solarbio (Beijing, China), was dissolved in dimethyl sulfoxide (DMSO, Solarbio) at a concentration of 20 mg/mL as the stock solution which was kept at -20 °C until use. Reactive oxygen species (ROS) detection kit (Cat number: CA1410), 2,3-bis (2-methoxy-4-nitro-5-sulfophenyl)-2H-tetrazolium-5-carboxanilide (XTT, Cat number: X2000) were also bought from Solarbio. Amphotericin B (AmB) was bought from Sangon (Shanghai, China). Propidium iodide (PI) was bought from Sigma-Aldrich (Shanghai, China). Bis-(1,3-dibutylbarbituric acid trimethine oxonol) [DiBAC_4_(3)] was bought from MCE company.

### 2.2 Determination of MIC and minimal fungicidal concentration

This assay was carried out through microdilution method recommended by CLSI-M27-A3. Wells of 96-well plate that were filled with only RPMI-1640 medium were used as blank control. Fungal cells from overnight cultures were collected through centrifugation (3000g, 5 min) and diluted in RPMI-1640 medium to obtain a density of 2×10^3^ cells/mL. 100 μL of such cell suspension was transferred into each well and 2×serial dilution was performed to get the final IBC concentrations from 80 μg/mL to 0.3125 μg/mL. To narrow down the MIC ranges of IBC, 64 μg/mL to 0.25 μg/mL of IBC were also tested in the MIC assays. AmB was used as positive control. Wells with cells but without IBC were used as negative control. DMSO in each well was less than 1% (v/v). The plates were incubated at 37 °C for 24 h statically. Then the fungal growth in well was inspected by naked eyes. The lowest concentration that caused no fungal growth was defined as MIC. The cultures in wells containing IBC concentrations equal or higher than MIC were transferred onto YPD agar plates which were incubated at 37 °C for 24 h. The lowest concentration that caused no colony grown on agar plates were set as MFC (Yang et al., 2018). In addition, to further quantify the effects of IBC on growth of *C. glabrata* strains, 3-(4, 5-dimethylthiazol-2yl)-2, 5-diphenyl-2H-tetrazoliumbromide (MTT) assay was performed at the end of MIC tests (Yang et al., 2018). Quality control strains used in this assay were *Candida krusei* ATCC 6258 and *Candida parapsilosis* ATCC 22019 (Guo et al., 2017).

### 2.3 Adhesion to polystyrene surfaces

*C. glabrata* ATCC2001 cells from overnight culture were diluted in 1640 medium (10^6^ cells/mL). Such suspensions were transferred into 96-well plates and exposed to different concentrations of IBC (1.25 μg/mL~10 μg/mL) at 37 °C for 1.5 h. After washing with PBS for three times, 100 μL XTT solution (0.5 mg/mL in PBS) were added. After a two-hour incubation in dark, the supernatants (70 μL) were transferred into a new plate and the optical density (OD) of each well at 490 nm (OD_490_) was detected with a multifunctional plate reader (Spark, Tecan, Switzerland) (Yang et al., 2024a).

Viability test was also performed after 1.5 h treatment to exclude the fungicidal effects of IBC on *C. glabrata* adhesion. In brief, all cells in each treatment were collected, diluted and spread on YPD agar plates. After 48-h incubation, the colony forming units (CFU) on each plate were counted.

### 2.4 Biofilms formation

Freshly prepared *C. glabrata* ATCC2001 cell suspension in 1640 medium (10^6^ cells/mL) was added into 96-well plates, followed by adding IBC (1.25 μg/mL~20 μg/mL) in a 2× serial dilution mode. Wells containing only medium were set as blank control while wells containing fungal suspension plus same volume of DMSO were set as negative control. After 24-hour growth in the presence of different combinations of IBC, the spent medium in each well was removed and 100 μL PBS and 10 μL MTS solution (CellTiter 96^®^ Aqueous One Solution Cell Proliferation Assay, Cat No: G3581, Promega, Beijing, China) were added. After a two-hour incubation in dark, the supernatants (70 μL) were transferred into a new plate and the OD_490_ of each well was detected with a multifunctional plate reader (Spark, Tecan, Switzerland). This assay was also performed on *C. glabrata* clinical isolates. The effects of IBC on biofilm biomass were also evaluated in 96-well plates, through crystal violet staining (Padmavathi et al., 2015). In brief, *C. glabrata* biofilms formed in the presence of IBC for 24 h and the spent medium in each well was removed. Biofilms were stained with 0.4% crystal violet for 15 minutes at 37 °C. After washing with distilled water for three times to remove excessive crystal violet, 100 μL 75% ethanol was added into each well and the plates were vortexed for 20s. Then, the OD_570_ of each well was determined with a multifunctional plate reader (Spark, Tecan, Switzerland). Wells with only medium were used as blank controls, which would be subtracted from OD_570_ of each well.

To directly show the effects of IBC on *C. glabrata* biofilm formation, biofilms formed in the presence of 0, 1.25, 2.5, 5, 10 and 20 μg/mL IBC were stained with fluorescent dye for cell wall Calcofluor White (CFW, final concentration: 20 μg/mL) for 10 minutes in dark. After washing with PBS for three times, biofilms were sent for confocal laser scanning microscope (CLSM, Olympus FV3000, Japan) analysis, which were performed in *xyz*-mode under a 40× objective. The step-size of scanning was 2 μm and the number of photo-sections were dependent on the height of biofilms. The obtained recordings of each biofilm were reconstructed into 3D pictures with Imaris 7.02.

### 2.5 Preformed biofilms

For tests on biofilm development, biofilms of *C. glabrata* strains were formed in the absence of any drugs for 24 h at 37 °C. Then biofilms were washed with PBS and exposed to fresh 1640 medium containing different concentrations of IBC (1.25 μg/mL ~20 μg/mL). 24 h later, the biofilms were subjected to MTS assay, as we mentioned above.

### 2.6 PI staining

The integrity of *C. glabrata* cells after IBC treatment was determined through PI staining (Yang et al., 2024b). *C. glabrata* ATCC2001 cells (10^6^ cells/mL) were treated with 0, 1.25, 2.5, 5 and 10 μg/mL IBC for 4 h at 37 °C. Then cells were stained with PI (10 μg/mL) for 15 minutes in the dark. After washing with PBS, fungal cells were subjected to CLSM (40× objective) for observation and analysis. In addition, the red fluorescence of PI in each group was also determined with a microplate reader at 535_nm_/617_nm_.

### 2.7 DiBAC_4_(3) staining

Another fluorescent dye DiBAC_4_(3) was used to evaluate the effects of IBC on cell membrane potential. *C. glabrata* cells treated with 0, 1.25, 2.5, 5 and 10 μg/mL IBC for 4h at 37 °C, were stained with DiBAC_4_(3) (final concentration: 2 μM). After washing with PBS, these cells were observed under CLSM. The cells with depolarized plasma membrane potential would emit green fluorescence.

### 2.8 ROS assay

The DCFH-DA-containing Solarbio ROS kit (SolarBio Company, Cat No: CA1410) was used to test whether IBC can cause ROS overproduction in *C. glabrata* cells. *C. glabrata* cells (10^6^ cells/mL) treated with 0, 1.25, 2.5, 5 and 10 μg/mL IBC for 4 h at 37 °C, were stained with DCFH-DA (20 μM) for 20 minutes in the dark. After PBS washing, fungal cells were observed under CLSM (40× objective). In addition, the fluorescence of ROS in each group was also determined with a microplate reader at Ex 485nm/Em 525nm.

### 2.9 N-acetyl-cysteine rescue tests

To test the influence of ROS on the IBC induced cell growth inhibition, NAC rescue tests were performed in the context of MIC assay. In brief, NAC was added in RPMI-1640 medium to obtain a final concentration of 5 mM. MIC assay was performed in RPMI-1640 medium and NAC-RPMI-1640 medium simultaneously, following the protocols described above (Part 2.2).

In addition, *C. glabrata* biofilms formed in the absence and presence of IBC, in the RPMI-1640 medium with or without containing 5 mM NAC, were subjected to MTS reduction assay.

### 2.10 Scanning electron microscope

*C. glabrata* ATCC2001 (10^6^ cells/mL in RPMI-1640 medium, in culture tubes) were treated with DMSO (control) or IBC (5 μg/mL) at 37 °C for 4 h, followed by centrifugation and fixation in 2.5% glutaraldehyde at 4 °C overnight. Then, samples were transferred to ServiceBio Company (Wuhan, China) for SEM scanning (Hitachi SU8100, Japan) under a magnification of 5,000. Samples were scanned in a high vacuum mode with a voltage of 3.0 kV.

### 2.11 Transmission electron microscope

*C. glabrata* ATCC2001 (10^6^ cells/mL in RPMI-1640 medium, in culture tubes) were treated with DMSO (control) or IBC (5 μg/mL) at 37 °C for 4 h, and then cells were collected by centrifugation (3000 g, 10 minutes) and fixed in 2.5% glutaraldehyde at 4 °C overnight. Afterwards, for TEM scanning, samples were sent to ServiceBio Company (Wuhan, China) where samples were desiccated, embedded and ultrathin-sectioned. Sectioned were stained with 2% uranyl acetate for 8 min and then with lead citrate for 8 min, and photographed subsequently with a Hitachi TEM system (HT7800, Hitachi, Tokyo, Japan) under a ×10,000 magnification.

### 2.12 Total nucleotide leakage

This assay was performed according to Campos et al, with slight modifications, to test the influence of IBC on the leakage of total nucleotide, which may also indicate the cell membrane damage (Campos et al., 2023). In brief, overnight cultured *C. glabrata* cells were resuspended in PBS and treatment with or without 5 μg/mL IBC for 8 h. After centrifugation at 10000 rpm for 10 minutes, the OD_260nm_ of the supernatant was recorded by a spectrophotometer (NanoDrop2000, Thermo Scientific, US).

### 2.13 Statistical analysis

All these assays abovementioned were performed in triplicate and repeated for at least three times. The data generated were expressed as mean ± SD and graphs were produced by GraphPad Prism 6.02. The analysis of variance was done by one-way ANOVA, followed by the Bonferroni or Dunnett comparison test. *p* < 0.05 indicated the statistical significance.

## 3 Results

### 3.1 Antifungal susceptibility of IBC

After 24-h incubation, the MIC of IBC against *C. glabrata strains* were inspected by naked eyes. As shown in **Table 1**, IBC had significant antifungal activities against the *C. glabrata* strains tested, with the MIC being from 4-5 μg/mL. The MFC of IBC against *C. glabrata* strains were also 5 μg/mL, except for clinical isolate 6, which was 8 μg/mL. The low ratio of MFC/MIC in these strains suggested that IBC exerted fungicidal effects on *C. glabrata* strains. As a positive control, AmB showed MIC of 1 μg/mL in these strains and isolates. The viability-concentration curves of IBC and AmB in these strains were shown in **Figure S1** and **Figure S2**.

**Table 1:** The antifungal activities of IBC against *Candida glabrata*.

Fungal strains	IBC	IBC	AmB	AmB
	MIC (μg/mL)	MFC (μg/mL)	MIC (μg/mL)	MFC (μg/mL)
*C. glabrata* ATCC 2001	5	5	1	2
*C. glabrata* clinical isolate 1	4	5	1	2
*C. glabrata* clinical isolate 2	4	5	1	2
*C. glabrata* clinical isolate 3	4	5	1	2
*C. glabrata* clinical isolate 4	4	5	1	2
*C. glabrata* clinical isolate 5	4	5	1	2
*C. glabrata* clinical isolate 6	4	8	1	2
*Candida krusei* ATCC 6258	4	4	1	2
*Candida parapsilosis* ATCC22019	4	4	1	2

### 3.2 Adhesion assay

Microbial adhesion was considered as the initial step for infection and biofilm formation. As shown in **Figure 1A**, IBC can significantly reduce the adhesion of *C. glabrata* ATCC 2001 cells to polystyrene surfaces. The inhibition of adhesion caused by 1.25-10 μg/mL IBC was from 29% to 85%, gradually. To test whether the adhesion inhibition was due to the fungicidal activity of IBC, the viable cells (measured by CFU) in each group were counted. As shown in **Figure 1B**, although 5 and 10 μg/mL IBC could significantly decrease the viable cells, indicating their fungicidal effects, 1.25 and 2.5 μg/mL IBC did not change the CFU numbers significantly. This, combined with **Figure 1A**, suggested that 1.25 and 2.5 μg/mL IBC did inhibit the adhesion of *C. glabrata* cells to polystyrene surfaces.

**Figure 1:**
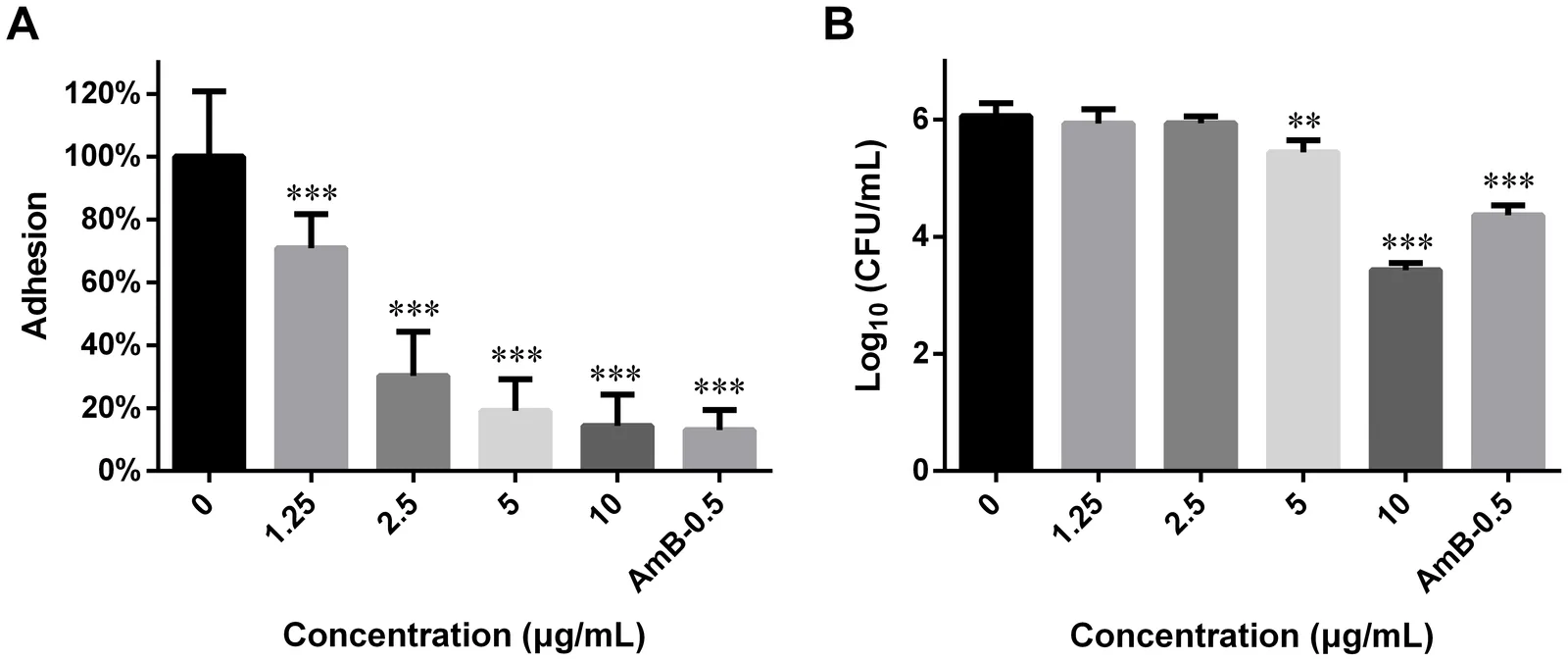
IBC inhibited the adhesion of *C. glabrata* ATCC 2001 to polystyrene surfaces. **(A)** Fungal cells treated with 0, 1.25, 2.5, 5 and 10 μg/mL IBC for 1.5 h, were washed with sterile PBS for three times and subjected to XTT assay. **(B)** the viable cells of each group were collected, diluted and spread on YPD agar plates. The CFU on each agar plate were counted. **p < 0.01, ***p < 0.001.

### 3.3 IBC inhibited the biofilm formation and development of *C. glabrata*

The effects of IBC on the biofilm formation of *C. glabrata* ATCC 2001 were determined through MTS assay in 96-well plates. As shown in **Figure 2A**, at concentrations below MIC, IBC can inhibit the biofilm formation of *C. glabrata* ATCC 2001. The metabolic activity of fungal biofilms exposed to 1.25 and 2.5 μg/mL IBC was reduced to 81% and 45%, as compared to IBC-free controls. 5- 20 μg/mL IBC can almost totally inhibit the biofilm formation, and the biofilm viability was around or below 20%, similar to the effect caused by 0.5 μg/mL AmB (**Figure 2A**). The results from biomass evaluation in *C. glabrata* ATCC 2001 showed a similar trend (**Figure 2B**). In another word, IBC could also inhibit the biomass of *C. glabrata* biofilms. In addition, in all the *C. glabrata* clinical isolates, IBC has also shown significant antibiofilm activity, as shown in **Figure 2C**. This kind of inhibition on biofilm formation was also confirmed in the 3D pictures of CFW-stained biofilms, which were obtained through CLSM scanning of biofilms and reconstructed with software. In these 3D graphs (**Figure 3**), biofilms formed in the absence of IBC had condensed structures and more cells. While with the increase in IBC concentration, the height of biofilm was lowered and the *Candida* cells were less. The space between cells was also enlarged by higher concentrations of IBC.

**Figure 2:**
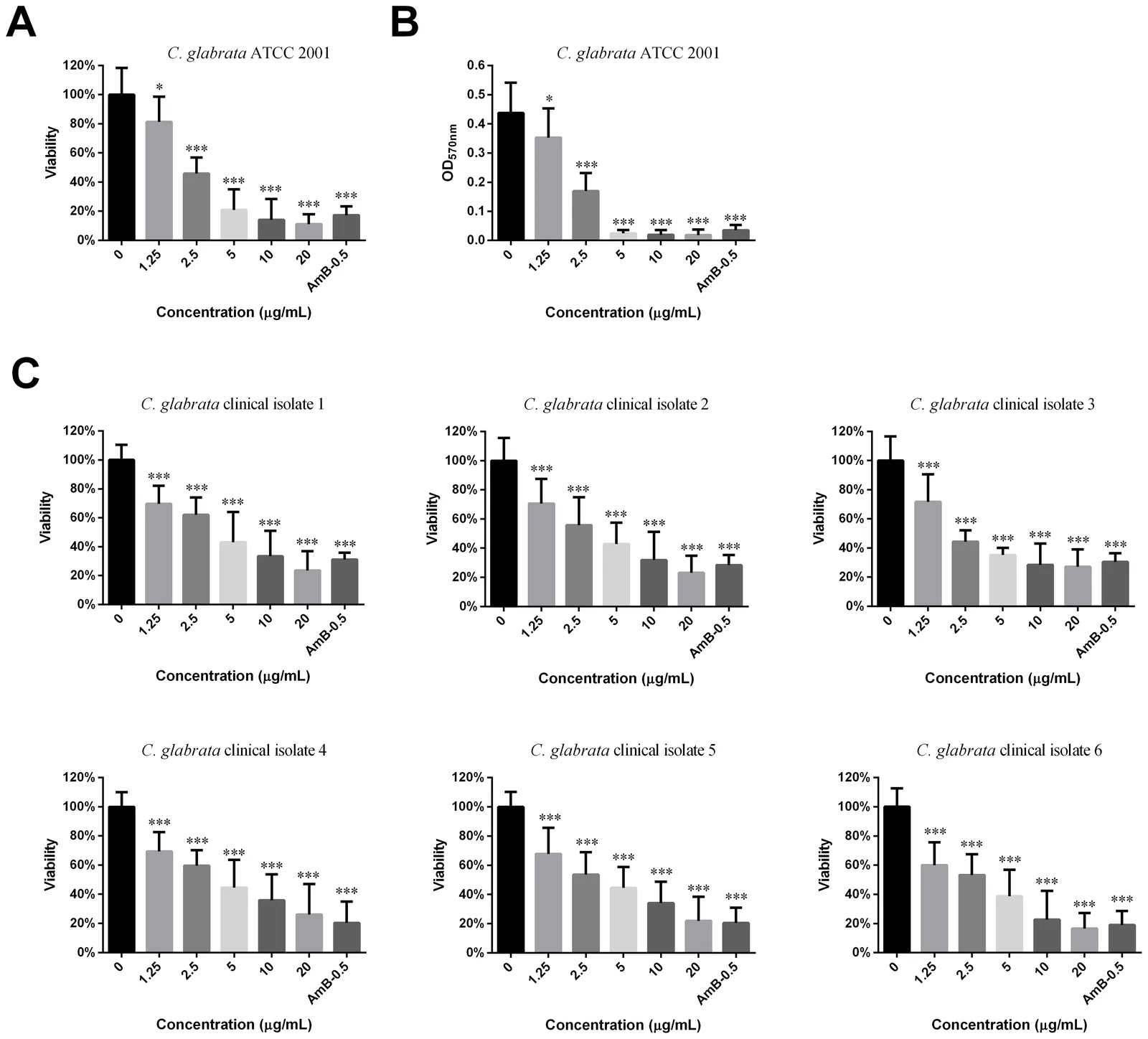
IBC inhibited the *C. glabrata* biofilm formation in standard strain ATCC 2001 **(A)** and clinical isolates **(C)**. Biomass of *C. glabrata* biofilms could also be inhibited by IBC **(B)**. Viability was determined through MTS reduction assays. Biomass was determined through crystal violet staining. *p < 0.05; **p < 0.01; ***p < 0.001.

**Figure 3:**
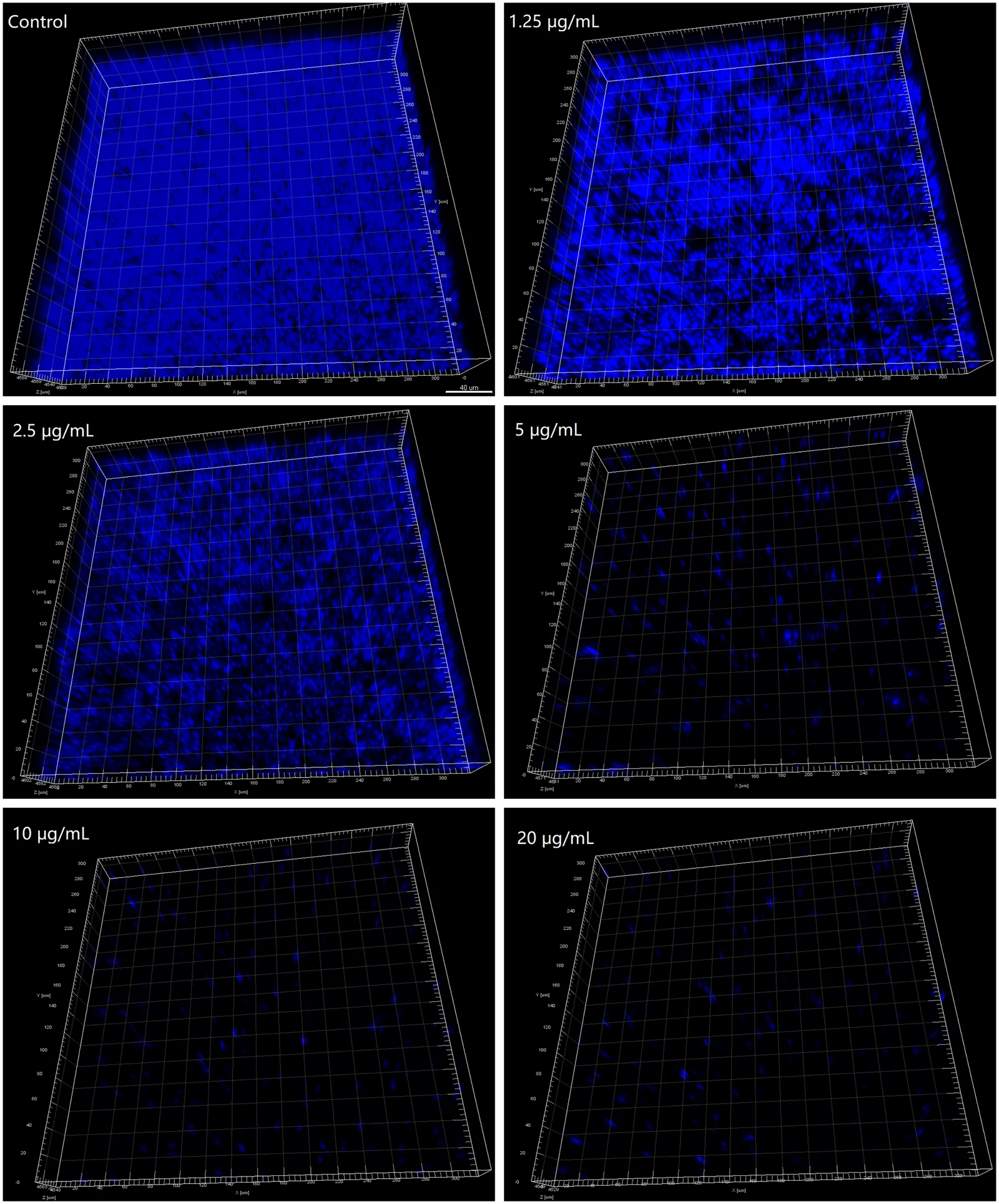
IBC inhibited the biofilm formation of *C. glabrata*. Biofilms formed in the presence of different concentrations of IBC were stained with CFW (20 μg/mL) for 10 minutes, washed with PBS, and then subjected to CLSM analysis using a 40× objective lens. The data obtained from *xyz*-mode recording were re-constructed with Imaris 7.2 to generate the three-dimensional pictures.

As for the preformed biofilm, the inhibitory effects of IBC were less potent than that on biofilm formation (**Figure 4**), which was similar to many antifungal agents. However, the decrease in the efficacy was limited in both standard strain and clinical isolates (**Figures 4A,B**). For example, 2.5 and 5 μg/mL IBC could inhibit the 24% and 54% viability of *C. glabrata* ATCC 2001 preformed biofilms, respectively (**Figure 4A**). The overall trend was similar to its effects on biofilm formation.

**Figure 4:**
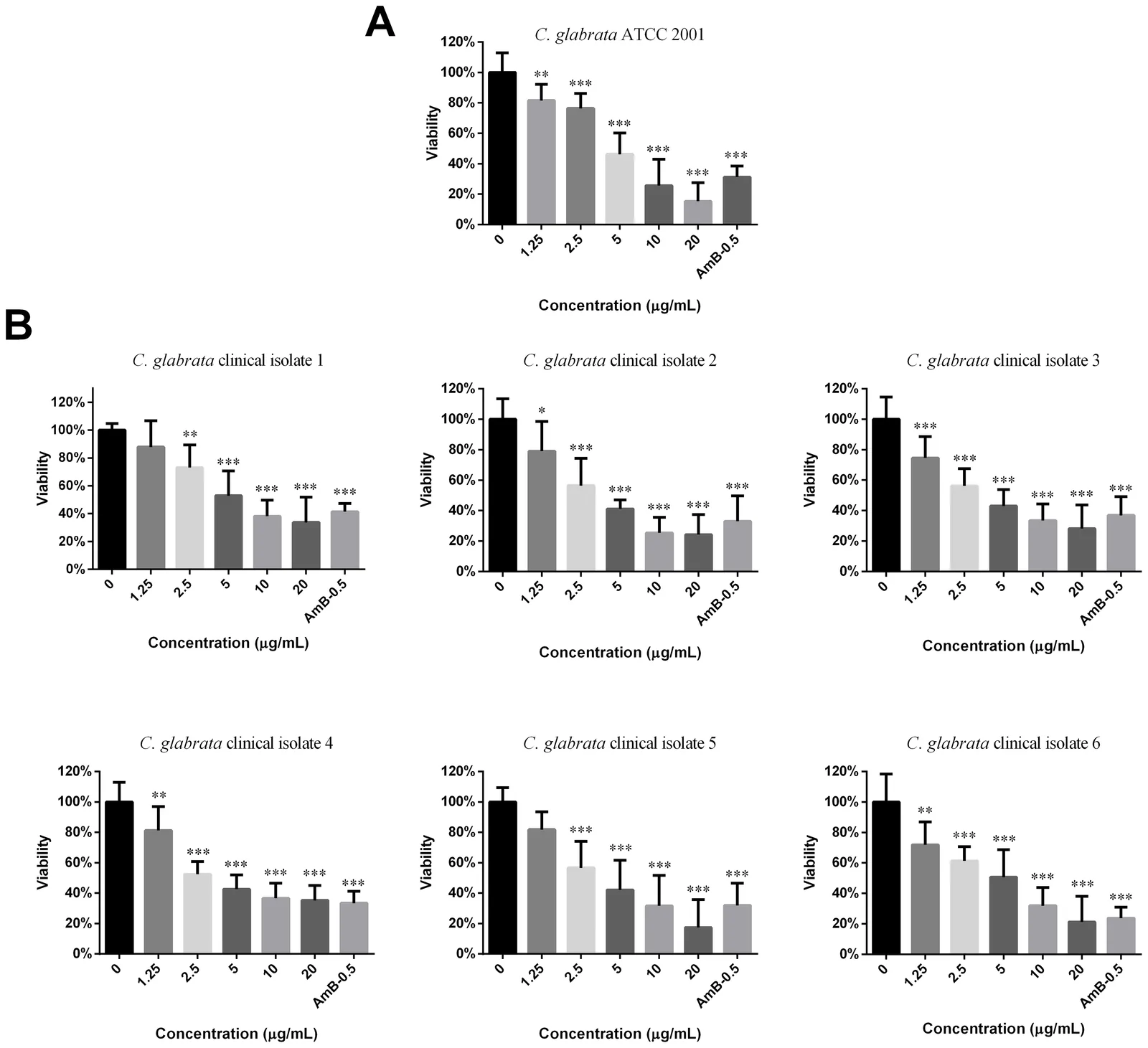
IBC inhibited the development of *C. glabrata* biofilms in standard strain ATCC 2001 **(A)** and clinical isolates **(B)**. Viability was determined through MTS reduction assays. *p < 0.05; **p < 0.01; ***p < 0.001.

### 3.4 IBC causes enhanced cell membrane permeability in *C. glabrata*

PI staining and subsequent CLSM photographing were employed to detect the membrane damages caused by IBC. As shown in **Figure 5A**, increasing the IBC concentration caused more *C. glabrata* cells to be stained with PI. This indicated the increased cell membrane permeability caused by IBC. In addition, PI staining were also determined through microplate reader. As shown in **Figure 5B**, the fluorescent intensity (relative fluorescence units, RFU) was increased as the IBC concentration rose. Although 1.25 and 2.5 μg/mL IBC did not cause a significant increase in RFU emitting from PI binding, the increase was true. When the IBC concentration went up to 5 and 10 μg/mL, the RFU increase became significant (**Figure 5B**). This was also consistent with the results from CLSM (**Figure 5A**).

**Figure 5:**
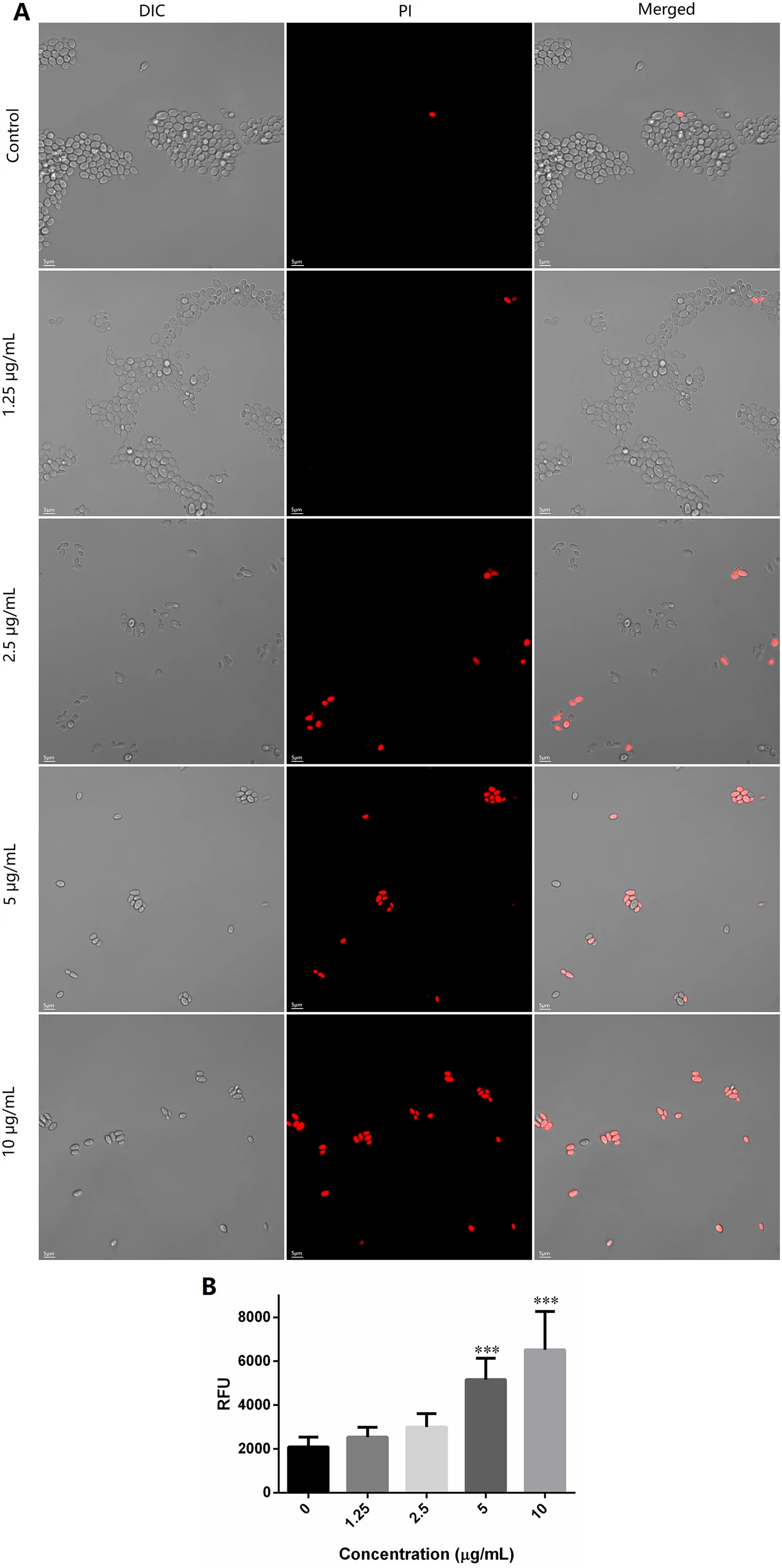
PI staining assays indicated that IBC increased the membrane permeability of *C. glabrata* cells. **(A)** Fungal cells were treated with different concentrations of IBC for 4 h, stained with PI and subjected to CLSM analysis. **(B)** Fungal cells were treated with different concentrations of IBC for 4 h, stained with PI and the fluorescent intensity was determined with a microplate reader (Spark, Tecan) at 535_nm_/617_nm_. ***p < 0.001.

### 3.5 IBC caused depolarization of cell membrane potential of *C. glabrata*

After the increase in cell membrane permeability was confirmed (**Figure 5**), cell membrane potential assay using DiBAC_4_(3) was also performed to further confirm the damages to cell membrane potential. As shown in **Figures 6A,B**, with the increase of the IBC concentration, the proportion of *C. glabrata* cells with green fluorescence increased. This indicated that IBC caused depolarization of cell membrane potential in *C. glabrata* cells.

**Figure 6:**
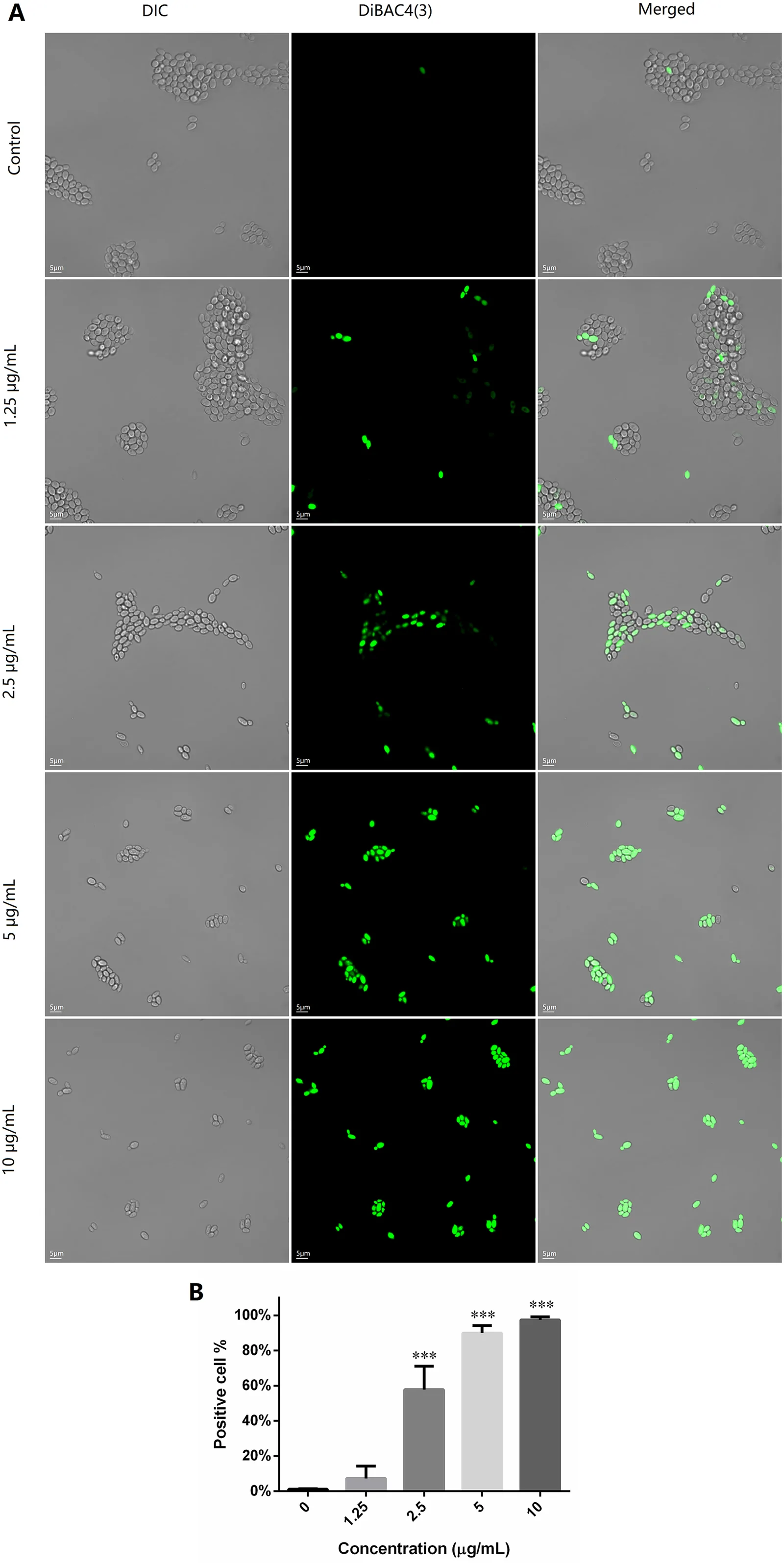
IBC caused alterations of cell membrane potential in *C. glabrata* ATCC 2001. **(A)** Fungal cells were treated with different concentrations of IBC for 4 h, stained with DiBAC_4_(3) and subjected to CLSM. **(B)** Statistics of DiBAC_4_(3)-positive cells in each group. ***p < 0.001.

### 3.6 IBC caused overproduction of ROS in *C. glabrata*

The intracellular ROS production was visualized through DCFH-DA staining and CLSM analysis. As shown in **Figures 7A, C***. glabrata* cells without IBC treatment had almost no green fluorescence, indicating very low ROS production. Increasing the concentration of IBC that *C. glabrata* cells were exposed to, more cells emitted green fluorescence. This meant that IBC caused excessive ROS production in *C. glabrata* cells. These microscopic results were further confirmed by the readings from microplate reader (**Figure 7B**).

**Figure 7:**
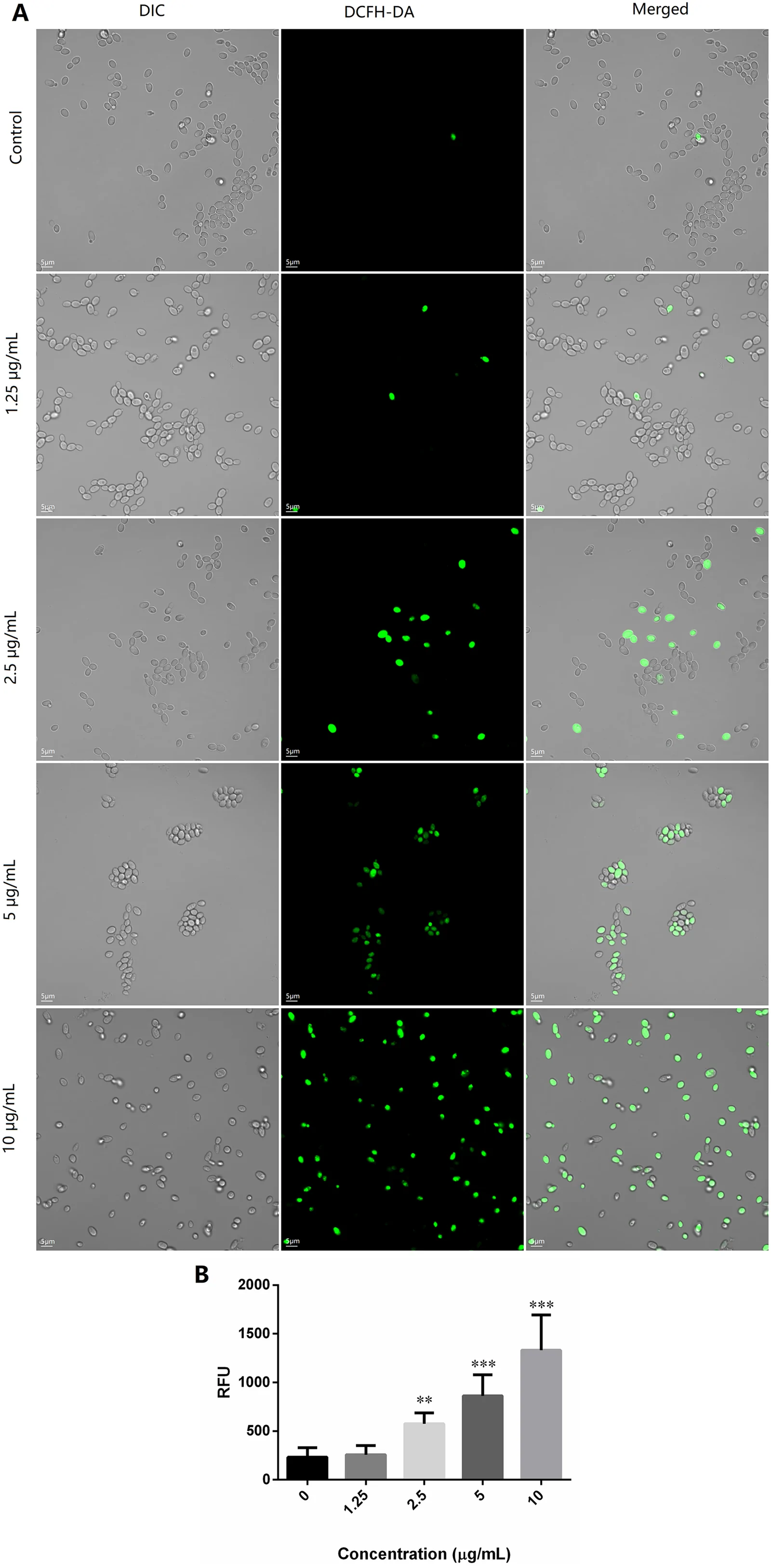
IBC induced excessive ROS production in *C. glabrata* cells. **(A)** Fungal cells treated with 0, 1.25, 2.5, 5 and 10 μg/mL IBC for 4 h were stained with DCFH-DA and analyzed by CLSM. **(B)** Fungal cells treated with 0, 1.25, 2.5, 5 and 10 μg/mL IBC for 4 h were stained with DCFH-DA, washed with PBS and detected with microplate reader at 485nm/525nm. **p < 0.01; ***p < 0.001.

### 3.7 NAC rescued the viability loss caused by IBC

To further investigate the role of ROS-inducing activity in the antifungal effects of IBC on *C. glabrata*, NAC rescue assays were performed. In planktonic cultures, 5mM NAC supplementation could increase the MIC of IBC in *C. glabrata* from 5 μg/mL to 10 μg/mL (**Figure 8A**).

**Figure 8:**
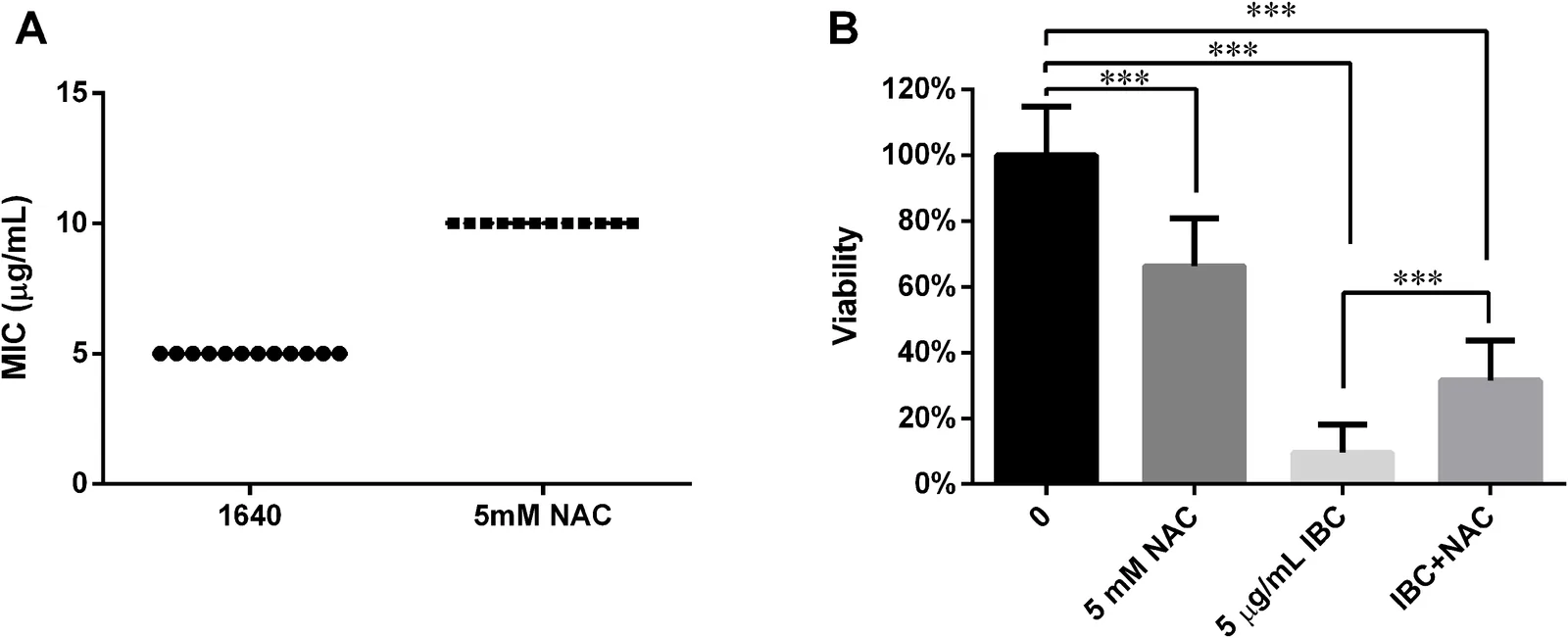
ROS production contributed to the antifungal and antibiofilm activities of IBC against *C. glabrata*. **(A)** NAC supplementation could increase the MIC of IBC against planktonic *C. glabrata* cells. **(B)** NAC supplementation could partly rescue the viability loss of *C. glabrata* biofilms caused by IBC. ***p < 0.001.

In *C. glabrata* biofilms, the addition of 5 mM NAC couldn`t increase the viability. In contrast, it decreased the viability of biofilms significantly (**Figure 8B**). But the addition of NAC could significantly rescue the viability loss of *C. glabrata* biofilms caused by 5 μg/mL IBC. These results indicated that ROS-inducing effects of IBC contribute to its antifungal and antibiofilm activities.

### 3.8 SEM and TEM

The effects of IBC on *C. glabrata* cells were also assessed with SEM and TEM. In micrographs from SEM, the cell surfaces of normal *C. glabrata* cells (without IBC treatment) were smooth (**Figure 9A**), while in cells exposed to 5 μg/mL IBC for 4h, the smooth surfaces were changed into wrinkled ones (**Figure 9B**). Even invaginated areas appeared on some cell surfaces and collapsed cells, which may indicate the efflux of intracellular materials, could also be found (**Figure 9B**).

**Figure 9:**
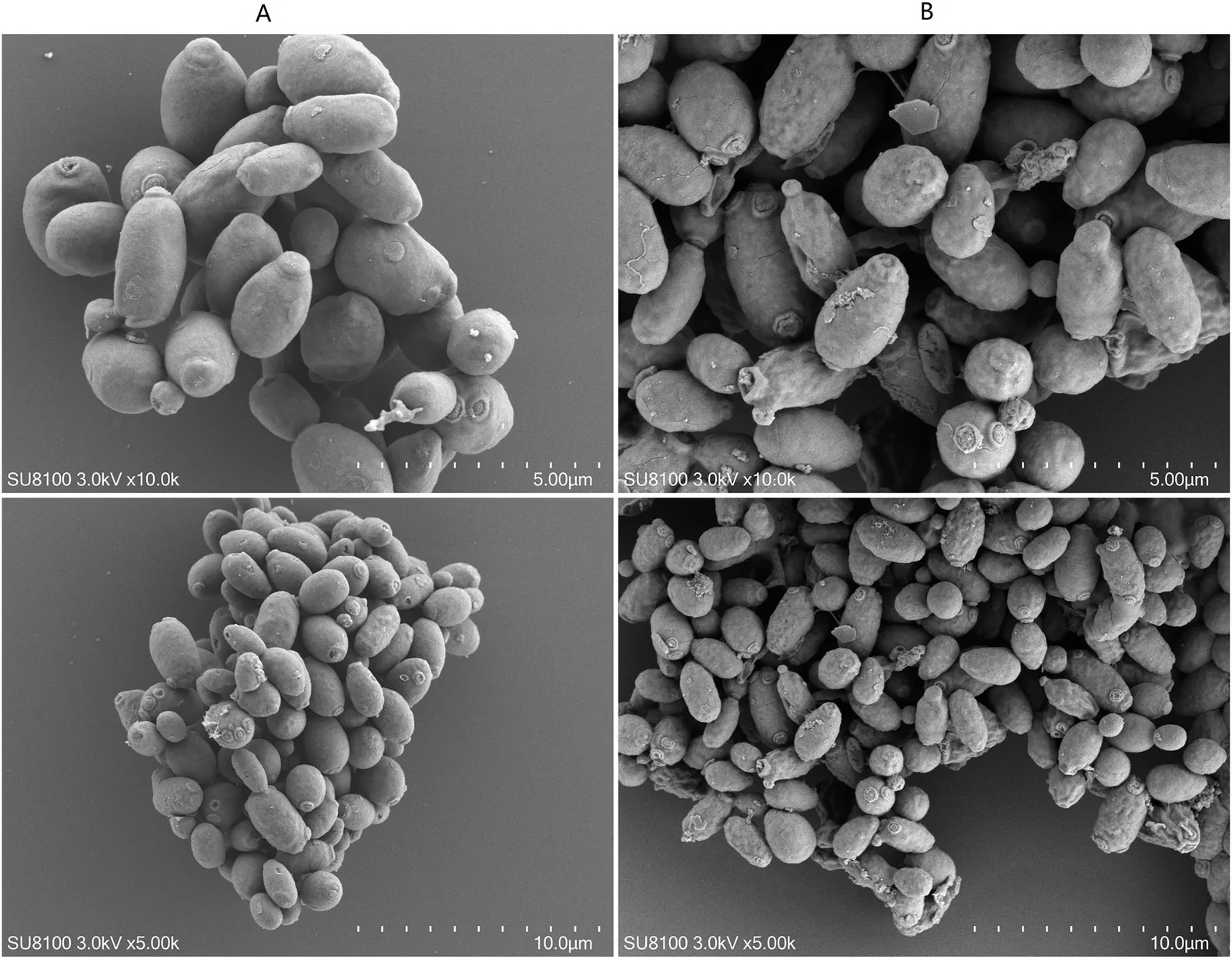
Effects of IBC on surface structures and morphologies of *C. glabrata* cells. **(A)** Normal *C. glabrata* cells. **(B)** *C. glabrata* cells treated with 5 μg/mL IBC for 4 h.

Morphological alterations of *C. glabrata* cell membrane after IBC administration were also evaluated through TEM. As shown in **Figure 10**, fungal cells grown in the absence of IBC had regular and complete cell membranes, while *C. glabrata* cells after IBC treatment for 4 h had damaged areas in the cell membrane. The disrupted integrity of cell membrane may lead to the death of *C. glabrata* cells.

**Figure 10:**
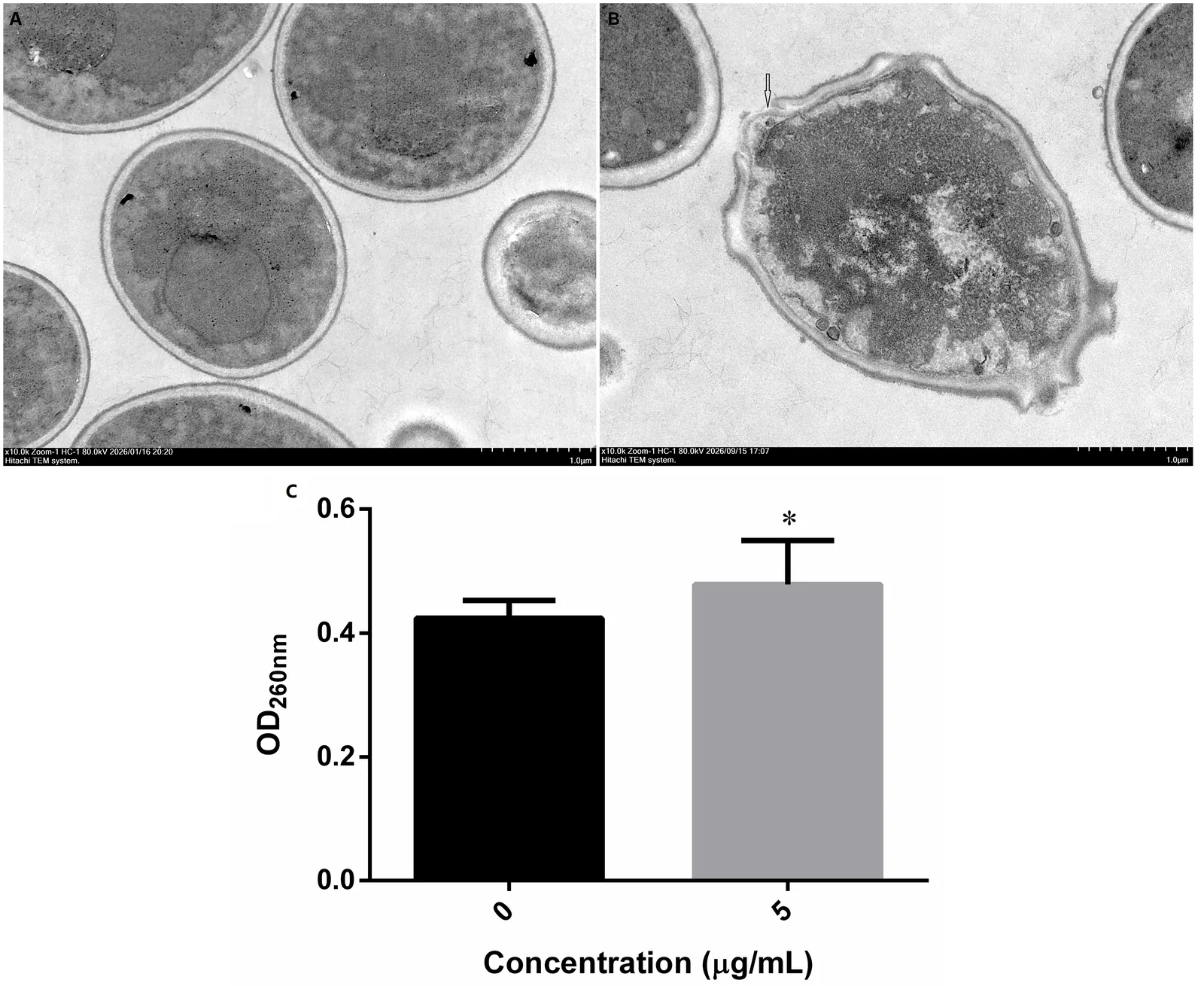
Ultrastructure of *C. glabrata* ATCC 2001 cells from TEM. **(A)** Normal *C. glabrata* cells. **(B)** 5 μg/mL IBC caused cell membrane and cell wall damages of *C. glabrata* cells. Cells were observed under TEM at 10,000× magnification. The hollow arrow indicated cell wall damages. **(C)** IBC treatment caused nucleotide leakage. *p < 0.05.

The release of intracellular components such as nucleotides into medium or buffer is also a sign of cell permeability change and cell membrane damage. After 8-hour treatment with 5 μg/mL IBC, the OD_260nm_ of supernatant was increased significantly (**Figure 10C**), as compared to control. The release of nucleotides caused by IBC also indicated the cell membrane damages.

## 4 Discussion

The past decades have seen an increase in the incidence of fungal infections (because of the growing susceptible individuals) and emergence of antifungal drug resistance (Liu et al., 2017; Shantal et al., 2022). *C. glabrata*, next to the critical priority pathogen *C. albicans*, has been listed as one of the high-priority pathogens by WHO (Katsipoulaki et al., 2024). *C. glabrata* can cause infections in bloodstream, oropharynx and urinary tract (Ahamad and Fatma, 2025; Ghosh et al., 2026). This fungus has many adhesion-related genes, can proliferate in phagocytes and form biofilms on mucosa and medical devices (d'Enfert and Janbon, 2016; Shantal et al., 2022; Ahamad and Fatma, 2025). The low intrinsic susceptibility of *C. glabrata* to fluconazole has caused emerging isolates that are resistant to this azole drug and the resistance to echinocandins is also growing in the past years (Shantal et al., 2022). This, and the paucity of antifungal drugs, call for the new antifungal agents.

Plants-derived small molecules have been attracting more interest from microbiologists and pharmacologists. Previously, we and other groups have shown that IBC have strong antifungal activities against *C. albicans* (Qian et al., 2024b; Wu et al., 2024; Yang et al., 2024a). The anti-*Candida* mechanisms involve damages to fungal cell membrane and cell wall, apoptosis induction, autophagy, suppression of enolase activity and glycolysis (Qian et al., 2024b; Wu et al., 2024; Yang et al., 2024a). Due to the distant phylogenetics from *C. albicans*, *C. glabrata* is renamed *Nakaseomyces glabratus* (Takashima and Sugita, 2022). The differences between *C. glabrata* and *C. albicans* in the susceptibility to fluconazole and their pathobiology (Takashima and Sugita, 2022), suggest one antifungal agent may have different performance and outcomes in these two fungal pathogens. Therefore, in this study, the activities of IBC against *C. glabrata* planktonic growth and biofilms were explored.

In our study, IBC had potent antifungal activities against *C. glabrata* (MIC = 4~5 μg/mL), both in standard ATCC strain and clinical isolates. This was consistent with the results from Mbaveng et al. (2008) and also extended their results in more strains. The strong inhibition of IBC on the adhesion, biofilm formation and development could also be seen in *C. glabrata*, similar to its effects on *C. albicans* (Qian et al., 2024b; Yang et al., 2024a). The inhibition of IBC on the preformed biofilms of *C. glabrata* was encouraging, since IBC can effectively inhibit the preformed biofilm at 2 MIC and above, while many antifungal agents have much higher concentrations required to inhibit the preformed biofilm by 50% (Yang et al., 2024b; Li et al., 2025). Therefore, IBC may be explored as an antibiofilm agent against *Candida* species.

We, and others, have shown that IBC could cause ROS in *C. albicans* (Yang et al., 2024a), *C. neoformans* (Qian et al., 2024a) and mammalian cells (Xiao et al., 2023), so we proposed that IBC may also induce ROS in *C. glabrata*. As proposed, IBC could cause excessive ROS production in *C. glabrata* cells, which was consistent with the studies mentioned above. NAC could elevate the MIC of IBC against C. glabrata, suggesting the role of ROS in the antifungal activity of IBC. The addition of ROS-scavenger NAC could rescue the viability loss caused by IBC in biofilms of *C. glabrata*, which further confirmed that the ROS overproduction contribute to the antibiofilm activity of IBC, at least partly. In fungal pathogens such as *C. albicans* and *C. neoformans*, IBC was shown to engender cell membrane damages (Qian et al., 2024b; Qian et al., 2024a; Yang et al., 2024a), and in bacteria such as *Clostridium difficile* (Liu et al., 2022) and *Staphylococcus aureus* (Assis et al., 2022), IBC can also disrupt the cell membrane integrity. In the Gram-negative *Klebsiella pneumoniae*, IBC was also shown to cause damages to the inner cell membrane (Geng et al., 2024). These indicated that in microbial pathogens, cell membrane damages may be one of the antibacterial and antifungal mechanisms of IBC. So, we tested the effects of IBC on *C. glabrata* cell membranes. Our PI staining and DiBAC_4_(3) staining results showed that IBC could increase the cell membrane permeability and depolarize the cell membrane potential, suggesting the damages to cell membrane integrity and function. This was consistent with previous publications (Qian et al., 2024b; Yang et al., 2024a). Data from TEM also showed the broken *C. glabrata* cell membrane caused by IBC. Micrographs obtained through SEM showed the rough cell surfaces and collapsed areas caused by IBC, which may also indicate the damages and loss of intracellular materials induced by IBC. SEM results may also suggest the alterations in cell wall of *C. glabrata*, since the cell wall is the outer layer of cell that provide rigidity. This was consistent with the results from other groups which showed that IBC caused cell membrane and cell wall damages in *C. albicans*, *C. neoformans* and *Magnaporthe grisea* (Liu et al., 2018; Qian et al., 2024b; Wang et al., 2024). The exact and detailed effects of IBC on *C. glabrata* cell membrane would be interesting and remain to be explored in the future.

## 5 Conclusion

In sum, the flavonoid IBC has potent activity against the planktonic growth of *C. glabrata*, with MIC of 4-5 μg/mL, in both standard strain and clinical isolates. IBC can also inhibit the biofilm formation and development of *C. glabrata* standard strain and clinical isolates. IBC causes excessive ROS production in *C. glabrata* cells, which may contribute to the damages to cell membrane. IBC treatment can significantly increase the permeability of cell membrane and cause the depolarization of cell membrane potential in *C. glabrata*. These changes, along with others, may eventually cause the death of fungal cells. Results from TEM and SEM also confirmed the cell membrane damages caused by IBC. Based on our results obtained here, we can report that IBC may be a promising candidate for treating fungal infections caused by *C. glabrata*, as well as other *Candida* species. However, animal experiments are required to confirm its *in vivo* efficacy, as well as its potential toxicity.

## Data Availability

The raw data supporting the conclusions of this article will be made available by the authors, without undue reservation.
