# Supplementary material for: The antifungal and antibiofilm effects of isobavachalcone on Candida glabrata (Nakaseomyces glabratus) are associated with intracellular ROS production and cell membrane damages
Source: Front Cell Infect Microbiol. 2026 Sep 29;16:1874660. doi: 10.3389/fcimb.2026.1874660 (PMC13658095; doi:10.3389/fcimb.2026.1874660)
Supplement: Supplementary file 1 [file Table1.docx]

Table S1. The clinical information of the six *Candida glabrata* isolates

|  | | *C. glabrata* clinical isolate 1 | *C. glabrata* clinical isolate 2 | *C. glabrata* clinical isolate 3 | *C. glabrata* clinical isolate 4 | *C. glabrata* clinical isolate 5 | *C. glabrata* clinical isolate 6 |
| --- | --- | --- | --- | --- | --- | --- | --- |
| Isolate/Access number | | JLUSH-2025-6160 | JLUSH-2025-6280 | JLUSH-2025-6336 | JLUSH-2025-6337 | JLUSH-2025-6446 | JLUSH-2025-6449 |
| Gender of patients | | Female | Male | Female | Female | Female | Male |
| Age (years) of patients | | 66 | 66 | 59 | 80 | 62 | 81 |
| Sample sources | | Blood | Blood | Blood | Blood | Blood | Blood |
| MIC of antifungals (μg/mL) | Fluconazole | 8 | 8 | 16 | 8 | 4 | 8 |
|  | Caspofungin | 1 | 0.12 | 0.06 | 1 | 0.25 | 0.5 |
|  | micafungin | 0.015 | 0.015 | 0.015 | 0.015 | 0.015 | 0.015 |
|  | anidulafungin | 0.03 | 0.06 | 0.015 | 0.03 | 0.03 | 0.03 |
|  | Itraconazole | 0.5 | 1 | 1 | 0.5 | 0.5 | 0.5 |
|  | Voriconazole | 0.25 | 0.25 | 0.25 | 0.25 | 0.25 | 0.25 |
